# Extremes of cerebral blood flow during hypercapnic squat‐stand maneuvers

**DOI:** 10.14814/phy2.15021

**Published:** 2021-10-07

**Authors:** Samuel C. Barnes, Victoria J. Haunton, Lucy Beishon, Osian Llwyd, Thompson G. Robinson, Ronney B. Panerai

**Affiliations:** ^1^ Department of Cardiovascular Sciences University of Leicester Leicester UK; ^2^ National Institute for Health Research (NIHR) Leicester Biomedical Research Centre University of Leicester Leicester UK

## Abstract

Squat‐stand maneuvers (SSMs) are a popular method of inducing blood pressure (BP) oscillations to reliably assess dynamic cerebral autoregulation (dCA), but their effects on the cerebral circulation remain controversial. We designed a protocol whereby participants would perform SSMs under hypercapnic conditions. Alarmingly high values of cerebral blood flow velocity (CBFV) were recorded, leading to early study termination after the recruitment of a single participant.

One healthy subject underwent recordings at rest (5 min sitting, 5 min standing) and during two SSMs (fixed and random frequency). Two sets of recordings were collected; one while breathing room air, one while breathing 5% CO_2_. Continuous recordings of bilateral CBFV (transcranial Doppler), heart rate (ECG), BP (Finometer), and end‐tidal CO_2_ (capnography) were collected.

Peak values of systolic CBFV were significantly higher during hypercapnia (*p* < 0.01), and maximal values exceeded 200 cm.s^−1^. Estimates of dCA (ARI) during hypercapnia were impaired relative to poikilocapnia (*p* = 0.03). The phase was significantly reduced under hypercapnic conditions (*p* = 0.03).

Here we report extremely high values of CBFV in response to repeated SSMs during induced hypercapnia, in an otherwise healthy subject. Our findings suggest that protocols performing hypercapnic SSMs are potentially dangerous. We, therefore, urge caution if other research groups plan to undertake similar protocols.

## Graphical abstract

Squat‐stand maneuvers are a popular method of inducing blood pressure oscillations to reliably assess dynamic cerebral autoregulation, but their effects on the cerebral circulation remain controversial. In order to better understand the cerebrovascular response to SSMs, and test the hypothesis that the changes in CBFV seen during repeated SSMs are indeed due to autoregulation, we designed a protocol whereby participants would perform repeated SSMs while breathing 5% CO_2_. We recorded alarmingly high values of CBFV, and therefore report our findings to inform other research groups who may be planning similar work.

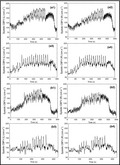

## 1 INTRODUCTION

Dynamic cerebral autoregulation (dCA) describes the acute changes in cerebrovascular resistance that control the volume of blood reaching the brain following changes in blood pressure (BP) (Paulson et al., 1990). Inadequate perfusion increases the risk of ischemia, whereas excessive perfusion poses the risk of arterial damage, disruption of the blood–brain barrier, and, in extreme cases, cerebral edema. (Euser & Cipolla, 2007).

In order to reliably assess dynamic cerebral autoregulation (dCA) using transfer function analysis (TFA), it is imperative to improve coherence by inducing oscillations in BP. One technique to achieve these oscillations is the repeated squat‐stand maneuver (SSM), which can be performed at either fixed‐frequency (FF) (Claassen et al., 2009; Smirl, Lucas, et al., 2014; Smirl et al., 2014b) or random‐frequency (RF) (Barnes et al., 2017). In recent years the SSM has become increasingly prominent, given its minimal equipment requirements, excellent tolerance and, above all, the high values of coherence it produces at the driven frequency (Brassard et al., 2017; Smirl et al., 2015).

While the peripheral physiology of the SSM is well understood (O'Donnell & McIlroy, 1962; Sprangers et al., 1991), relatively little is known about the response of the cerebrovasculature. One study has demonstrated that the cerebrovascular response to SSMs is dominated by the influence of BP, which is to be expected given the underlying physiology (Barnes et al., 2017). However, there was also a larger than expected metabolic component, which suggests that other physiological mechanisms may be contributing to the changes in CBFV.

It is well‐documented in the literature that dCA is impaired during hypercapnia and improved during hypocapnia (Aaslid et al., 1989; Birch et al., 1995; Paulson et al., 1990). One previous study has performed SSMs under hypocapnic, normocapnic and hypercapnic conditions (Birch et al., 1995), and found that phase (the time delay between the BP and CBFV waveforms) was enhanced in hypocapnia and reduced in hypercapnia, suggesting improved and impaired dCA, respectively. Another study used SSMs to analyze the cerebrovascular response to hypercapnia in the context of ischemic pre‐conditioning. Again, dCA was shown to be impaired in hypercapnic conditions (Carter et al., 2020). However, maximal values of CBFV were not reported in either study.

It also appears SSMs may partially impair dCA; whether this is due to the increased pCO_2_ during these maneuvers or due to the maneuvers themselves remains unclear (Barnes, Ball, Haunton, Panerai, et al., 2017). In order to better understand the cerebrovascular response to SSMs, and test the hypothesis that the changes in CBFV seen during repeated SSMs are, indeed, due to autoregulation, we designed a protocol whereby participants would perform repeated SSMs while breathing 5% CO_2_. However, as we will describe below, the values of CBFV elicited during this study were extremely high, and the study was, therefore, terminated after the evaluation of a single participant. Given our concerns regarding the safety of using this concentration of inhaled CO_2_ in repeated SSMs, we report our data to inform research groups who may be planning similar research.

## 2 MATERIALS AND METHODS

The study was carried out according to the latest approved protocol, International Conference on Harmonization‐Good Clinical Practice (ICH‐GCP), relevant regulations, and standard operating procedures as well as in accordance with the Declaration of Helsinki. The study had ethical approval (University of Leicester ethics reference 8442‐vjh12‐cardiovascularsciences), and the participant provided written informed consent. Data were handled in accordance with the UK Data Protection Act and General Data Protection Regulations.

### 2.1 Experimental procedures

The experiment was performed in a well‐lit, environmentally controlled laboratory that was free from distraction and kept at a temperature of 20–24⁰C. The participant avoided strenuous exercise, caffeine, smoking, large meals, and alcohol in the 4 h prior to their visit.

### 2.2 Instrumentation

Heart rate was measured using three‐lead ECG. A tilt‐sensor was attached to the subject's right thigh 20 cm above the superior border of the patella to measure the angle of the squatting motion. Doppler probes (2MHz, Viasys Companion III) were placed over the left and right temporal windows, and were held in a constant position at a fixed angle by a custom‐built headset to measure CBFV in the middle cerebral arteries (MCA). Nasal capnography (Salter labs, ref 4000) was used to measure end‐tidal CO_2_. Beat‐to‐beat estimates of BP were obtained through arterial volume‐clamping of the digital artery (Finometer, FMS); this method being shown to accurately reflect intra‐arterial BP changes (15, 21). The servo‐reset mechanism was disabled throughout the recordings to allow for a continuous BP trace, but enabled between recordings. The right hand was held in position with a sling to minimize movement throughout the recordings, and to keep the finger cuff at the height of the heart. Finally, intermittent brachial BP was measured using a validated electrosphygmomanometer (UA 767 BP monitor) to calibrate the Finometer recordings. A gas mixture of 5% CO_2_, 21% O_2_, and 76% N_2_ was breathed through a twin‐port face mask, affixed to the head by a strap (Intersurgical).

Continuous analog recordings were digitized at 500 samples/s by a Physiological Data Acquisition System (PHYSIDAS) designed by the Leicester Medical Physics Department for subsequent analysis.

### 2.3 Poikilocapnia

One set of recordings were obtained under poikilocapnic conditions (Barnes, Ball, Haunton, Panerai, et al., 2017). Following a 10‐min period of rest and stable recordings, four recordings were performed. The recordings were as follows: a 5‐min baseline recording of the patient sitting quietly with their eyes open; a 5‐min baseline recording of the patient standing quietly with their eyes open; FFSSMs (15 squats at a frequency of 0.05 Hz, preceded and followed by 90s standing); RFSSMs (15 squats of random duration with random periods of standing between them, preceded and followed by 90s standing). Both sets of SSMs were guided by a set of computer‐generated visual cues, and the duration of the RFSSMs was determined by a custom‐designed computer program (Barnes, Ball, Haunton, Panerai, et al., 2017).

### 2.4 Hypercapnia

Following a 10‐min period of rest and stable recordings, four further recordings were performed. The recordings were as follows: an 8‐min baseline recording of the patient sitting quietly with their eyes open; an 8‐min baseline recording of the patient standing quietly with their eyes open; FFSSMs (15 squats at a frequency of 0.05 Hz, preceded and followed by 90s standing); RFSSMs (15 squats of varying duration identical to the poikilocapnic RFSSMs, preceded and followed by 90s standing). The twin‐port face‐mask was worn for the entire duration of each of these four recordings, but was removed between recordings to allow the participant to breathe normally.

During the sitting and standing recordings, the subject began by breathing room air for one minute before breathing the 5% CO_2_ gas mixture for five minutes, followed by a further two minutes of room air to make an 8‐minute recording. For the two SSMs, the subject breathed room air for the first minute of the recording, before switching to the 5% CO_2_ gas mixture for a further minute. Once the 15 maneuvers were completed the subject breathed room air for two minutes until the end of the recording.

Between all four recordings, the subject was given a minimum of five minutes to recover from the effects of hypercapnia.

### 2.5 Data analysis

The readings from the Finometer were calibrated to the brachial BP recordings. Data were visually inspected; non‐physiological spikes in CBFV were removed through linear interpolation. Subsequently, all signals were filtered in the forward and reverse direction using an eighth‐order Butterworth low‐pass filter with a cut‐off frequency of 20 Hz. The beginning and the end of each cardiac cycle were detected in the BP signal, and mean values of BP, CBFV, and heart rate were obtained for each heartbeat. Beat‐to‐beat parameters were interpolated with a third‐order polynomial and resampled at 5 Hz to generate signals with a uniform time base.

Using the inverse fast Fourier transform, the CBFV response to a step change in BP was also derived (Panerai et al., 1998). The CBFV step response was compared with 10 template curves proposed by Tiecks et al. (Tiecks et al., 1995) and the best fit curve corresponded to the ARI. A new procedure was adopted using the normalized mean square error for fitting the Tiecks model to the CBFV step response and a minimum threshold for the coherence function (0.15–0.25 Hz) to accept or reject estimates of ARI (Panerai et al., 2016).

dCA was modeled using transfer function analysis (TFA), using mean BP as input and corresponding changes in CBFV as output as described previously (Giller, 1990; Panerai et al., 1998; Tzeng et al., 2012; Zhang et al., 1998). The Welch method was adopted for smoothing spectral estimates obtained with the fast Fourier transform (102.4 s segments, 50% superposition) leading to frequency dependent estimates of coherence, gain, and phase. For FFSSMs, point estimates of coherence, phase, and gain were calculated at 0.05 Hz (6, 29). For RFSSMs, estimates were averaged for the very‐low (VLF, 0.02–0.07 Hz), low (LF, 0.07–0.20 Hz), and high (HF, 0.20–0.50 Hz) frequency ranges (Claassen et al., 2016).

In order to account for the discrepancy in file length, and to ensure that we were comparing periods of poikilocapnia with periods in the hypercapnia files where the subject was loaded with CO_2_, we analyzed discrete segments of the recordings. Specifically, for sitting and standing, we analyzed the period of 60–300 s. For FFSSMs this was 60–480 s, and for RFSSMs this was 60–440 s.

### 2.6 Statistical analysis

Hemispheric values for CBFV, ARI, and TFA were compared using paired t‐tests. Where no significant differences existed, these data were averaged for further analysis. Mean values of physiological parameters during hypercapnia and poikilocapnia were compared with repeated‐measures ANOVA, using exposure to 5% CO_2_ as the between‐effect. Significance was set at *p* < 0.05.

## 3 RESULTS

### 3.1 Subject

We recruited one healthy volunteer, a 22‐year‐old male. The participant was cardiovascularly well‐trained, performing moderate‐high intensity exercise four times a week.

### 3.2 Participant response to induced hypercapnia

**FIGURE 1:**
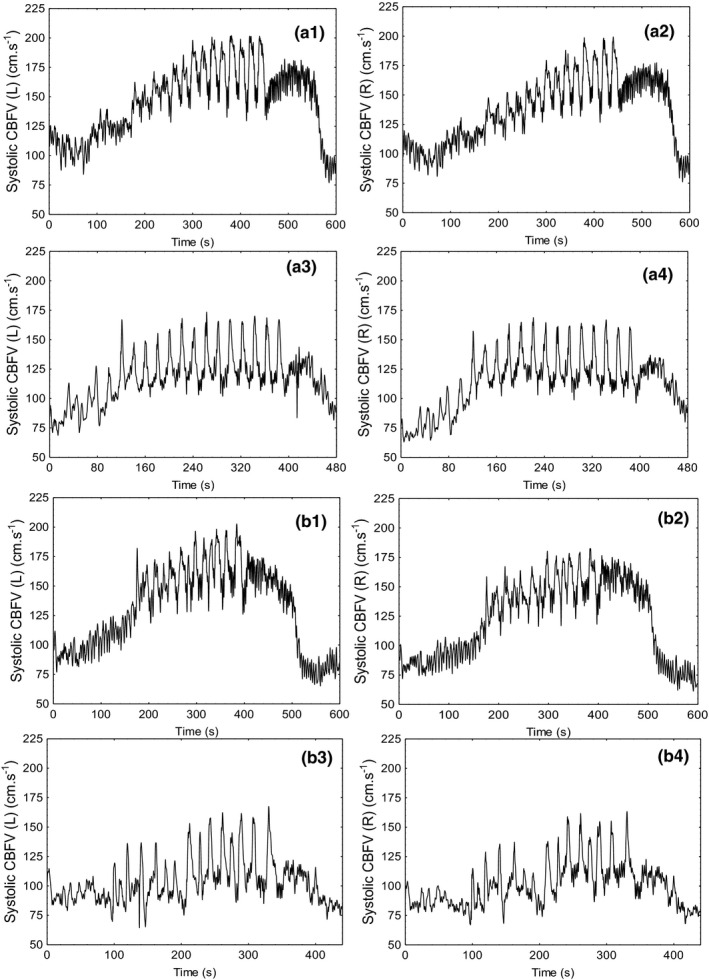
Response of systolic CBFV to FFSSMs (a) during hypercapnia (a1–a2) and poikilocapnia (a3–a4), and RFSSMs (b) during hypercapnia (b1–b2) and poikilocapnia (b3–b4). L, left; R, right

The response of physiological parameters to the four recordings during poikilocapnia and hypercapnia is detailed in Table 1. There were no significant differences in cerebrovascular parameters between the left and right MCA. During the hypercapnic SSMs, mean CBFV was significantly increased compared to the same values obtained during poikilocapnia (*p* = 0.03). Peak values of systolic CBFV were significantly higher under the influence of hypercapnia (*p* < 0.01), and maximal values were extremely high (Figure 1), exceeding 200 cm.s^−1^. As expected, end‐tidal CO_2_ was significantly elevated following the inhalation of CO_2_ (*p* < 0.01). Under hypercapnic conditions, average end‐tidal CO_2_ was elevated during FF and RFSSMs compared to their respective baseline recordings of the subject sitting and standing (*p* < 0.01). Following the maneuvers in the hypercapnic state, the participant reported light‐headedness and a moderate headache. No such symptoms were noted following poikilocapnic SSMs.

**TABLE 1:** Distribution of cerebrovascular and systemic parameters for different postures and maneuvers for poikilocapnia (P) and hypercapnia (H) for the single experimental subject

Parameter	CO_2_	Seated	Standing	FFSSM	RFSSM
EtCO_2_ (mmHg)	P	43.5 ± 1.4	39.6 ± 1.1	43.8 ± 3.0	41.5 ± 3.0
	H	46.9 ± 1.2	48.1 ± 2.4	54.7 ± 4.4	54.3 ± 4.6
Systolic BP (mmHg)	P	141.3 ± 6.2	141.1 ± 11.3	129.5 ± 19.2	119.4 ± 13.5
	H	130.2 ± 7.9	137.9 ± 8.4	150.1 ± 18.7	157.9 ± 22.7
Diastolic BP (mmHg)	P	86.7 ± 3.7	92.3 ± 3.9	84.2 ± 14.3	90.3 ± 9.3
	H	82.7 ± 3.1	93.0 ± 4.8	96.6 ± 12.8	81.8 ± 14.0
Heart rate (bpm)	P	70.2 ± 10.1	94.9 ± 7.7	105.7 ± 20.6	111.4 ± 17.3
	H	87.2 ± 8.5	102.7 ± 8.8	114.1 ± 18.5	116.6 ± 15.6
Mean CBFV^a^ (cm.s^−1^)	P	63.4 ± 3.4	59.9 ± 4.9	69.9 ± 21.3	60.4 ± 20.1
	H	74.8 ± 5.7	72.4 ± 8.3	99.2 ± 23.9	95.8 ± 23.3
Maximal systolic CBFV (cm.s−^1^)	P	117.8	106.5	168.4	165.4
	H	132.2	135.6	200.4	192.3
Coherence	P	0.31 ± 0.02	0.38 ± 0.01	0.98 ± 0.02	0.96 ± 0.01
	H	0.44 ± 0.03	0.81 ± 0.11	0.97 ± 0.01	0.97 ± 0.01
Gain (cm.mmHg^−1^s^−1^)	P	0.80 ± 0.06	1.30 ± 0.13	1.71 ± 0.07	2.90 ± 0.14
	H	1.06 ± 0.09	1.50 ± 0.21	1.39 ± 0.01	1.40 ± 0.04
Phase (rad)	P	1.41 ± 0.37	1.17 ± 0.14	0.52 ± 0.01	0.51 ± 0.04
	H	0.63 ± 0.08	0.28 ± 0.11	−0.02 ± 0.05	0.08 ± 0.04
ARI	P	5.4 ± 0.01	6.0 ± 0.03	3.9 ± 0.21	4.0 ± 0.14
	H	4.8 ± 0.45	2.9 ± 0.05	1.9 ± 0.06	1.8 ± 0.14

Mean ± within‐subject SD values for cerebral hemodynamic and transfer function analysis parameters correspond to the average of measurements from the right and left MCA.

Abbreviations: ARI, autoregulation index; BP, blood pressure; CBFV, cerebral blood flow velocity; EtCO_2_, end‐tidal CO_2_; FFSSM, fixed frequency squat stand maneuver; RFSSM, random frequency squat stand maneuver.

^a^ CBFV averaged across hemispheres (Barnes, Ball, Haunton, Panerai, et al., 2017).

### 3.3 Metrics of autoregulation

**FIGURE 2:**
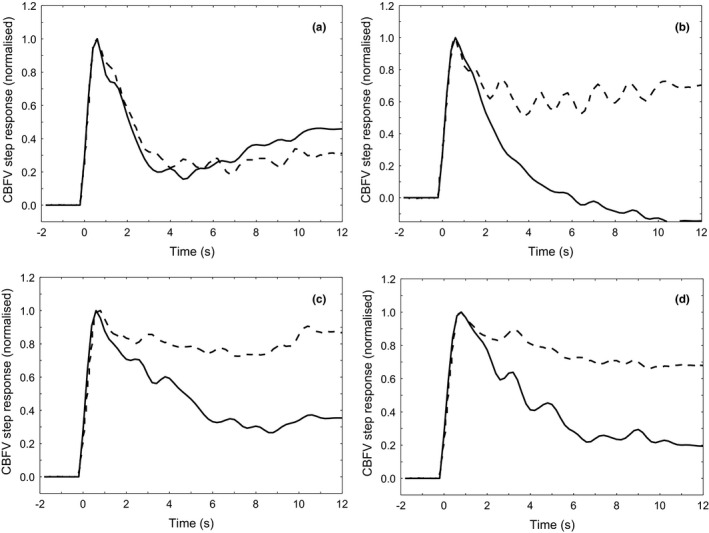
CBFV step responses for poikilocapnia (solid line) and 5% CO2 (dashed line) during (a) seating, (b) standing, (c) fixed frequency SSM, (d) random frequency SSM

Figure 2 gives the step responses following the changes in CBFV induced by the SSMs. The ARI during hypercapnia was significantly impaired relative to poikilocapnia (*p* = 0.03). While estimates of coherence (*p* = 0.21) and gain (*p* = 0.32) were comparable, the phase was significantly reduced under hypercapnic conditions (*p* = 0.03).

Estimates of transfer function are given in Table 1. Coherence values for both fixed‐frequency and random‐frequency maneuvers were very high, in keeping with previous studies (Barnes, Ball, Haunton, Panerai, et al., 2017; Brassard et al., 2017; Claassen et al., 2009; Smirl et al., 2015). Estimates of phase and ARI were both reduced during states of hypercapnia for both fixed and random‐frequency maneuvers relative to poikilocapnia.

## 4 DISCUSSION

To our knowledge, there is no definitive “safe” upper limit for systolic CBFV, and we are unaware of any studies which have attempted to quantify this. However, in comparison to previous studies which have employed the SSM (Barnes, Ball, Haunton, Panerai, et al., 2017; Smirl et al., 2015), the mean and peak values of hypercapnic CBFV reported here are remarkably high. In the context of impaired dCA, as demonstrated by the ARI, there is a theoretical increased risk of hemorrhage and microvascular damage (Iadecola & Davisson, 2008). This is of great relevance to researchers employing these techniques and raises questions about the safety of SSMs under intensely hypercapnic conditions, particularly in elderly or non‐healthy populations.

SSMs have previously been performed under hypercapnic conditions in two studies, yet in neither study were maximal velocities of CBFV reported (Birch et al., 1995; Carter et al., 2020). As demonstrated in Figure 1, systolic CBFV values exceeded 200 cm.s^−1^ during SSMs performed under hypercapnic conditions. Notably, during hypercapnia, CBFV climbed consecutively with each maneuver. This could be an exercise effect directly impairing dCA (Barnes, Ball, Haunton, Panerai, et al., 2017; Batterham et al., 2020), yet no such trend is noted in the poikilocapnic state in our single participant.

### 4.1 Physiological parameters

Carter et al. reported an average end‐tidal CO_2_ of 46.6 ± 2.7 mmHg in their participants (Carter et al., 2020), who also breathed 5% CO_2_. Clearly, this is a large discrepancy compared to our reported values of 54.7 ± 4.4 mmHg during FFSSMs and 54.3 ± 4.7 mmHg during RFSSMs, so the “dose” of CO_2_ received by our participant may account for the large reduction in ARI and subsequent high velocities of CBFV. The reason for this discrepancy is unclear, and indeed the dose–response relationship between CO_2_ and impaired dCA is not yet completely understood. Minhas et al. studied the dose–response relationship in the physiological range and generated an EtCO_2_/CBFV response curve (Minhas et al., 2018), but their range did not include the values reported here. Their study reported mean maximal EtCO_2_ values of 47.9 mmHg in participants during induced hypercapnia while resting supine.

As highlighted previously, SSMs may produce an “exercise effect” in addition to their effect on BP (Barnes, Ball, Haunton, Panerai, et al., 2017; Batterham et al., 2020). As the SSM is an active maneuver, it likely leads to an increased oxygen requirement, particularly when undertaken repeatedly; this is supported by the increased HR during both poikilocapnic and hypercapnic SSMs (Table 1). In our participant, who was well‐trained and breathing 5% CO_2_, attempts to meet this oxygen requirement through breathing the gas mixture may have led to an increased absorption of CO_2_. Given our participant's exercise capacity, it is possible that they inhaled large tidal volumes of 5% CO_2_ during the SSMs, leading to the levels of end‐tidal CO_2_ reported here.

### 4.2 Limitations

The key limitation here is that these data were obtained from one participant, during a single episode of hypercapnia. After these data were collected and analyzed, it was felt unsafe to proceed with further participants due to the exceedingly high values of systolic CBFV recorded.

Cerebral autoregulation has been shown to be impaired in young athletes (Labrecque et al., 2017, 2019). Our participant was cardiovascularly well trained, but a formal assessment of his fitness with VO2 max was not performed. However, while the values of ARI reported during hypercapnia are very low, the paired poikilocapnic values indicate that this is unlikely to be a participant effect.

Finally, the extreme values of end‐tidal CO_2_ reported during hypercapnic SSMs may have caused vasodilation of the insonated arteries, leading to underestimates of CBF (Willie et al., 2011). It is, therefore, feasible that, despite the extremely high systolic CBFV values recorded, the true flow was even higher.

## 5 CONCLUSION

In summary, we report extremely high values of mean and peak systolic CBFV in response to repeated SSMs during induced hypercapnia, in an otherwise healthy subject. SSMs and hypercapnia have both been shown to individually impair cerebral autoregulation. The values of CBFV obtained here suggest that protocols performing SSMs during hypercapnia induced by 5% CO_2_ are potentially dangerous. We, therefore, urge caution if other research groups plan to undertake similar protocols. Future work employing a similar protocol and a reduced concentration of CO_2_ may be safer and may facilitate an answer to the initial research question.

## CONFLICTS OF INTEREST

None to declare.

## AUTHOR CONTRIBUTIONS

TGR, RBP, and VJH conceived and designed the research; SCB, LB, and OLL performed experiments; SCB, RBP, and LB analyzed data; SCB and RBP interpreted the results of data; SCB and RBP prepared figures and drafted manuscript; SCB, LB, RBP, and VJH edited and revised manuscript. All authors approved the final version of the manuscript.
